# Safety and Effectiveness of a Peripheral Rheolytic Thrombectomy Catheter in ST-Segment Elevation Myocardial Infarction: A Case Series

**DOI:** 10.3390/jcdd12020072

**Published:** 2025-02-14

**Authors:** Giuseppe Giacchi, Agnese Bentivegna, Ida Logatto, Antonino Nicosia

**Affiliations:** Cardiology Department, Cardio-Neuro-Vascular Institute, Giovanni Paolo II Hospital, ASP 7 Ragusa, 97100 Ragusa, Italy; agnese.bentivegna@asp.rg.it (A.B.); ida.logatto@asp.rg.it (I.L.); antonino.nicosia@asp.rg.it (A.N.)

**Keywords:** rheolytic thrombectomy, mechanical thrombus aspiration, thrombotic coronary lesions, ST-segment elevation myocardial infarction, STEMI, acute coronary syndromes (ACS)

## Abstract

Percutaneous treatment of highly thrombotic coronary lesions is demanding, due to worse acute and long-term clinical outcomes. In this report, we describe a case series of six patients with ST-segment elevation myocardial infarction and high-thrombus-burden coronary lesions. All patients were treated with the AngioJet Solent^®^ Dista catheter, a rheolytic thrombectomy device designed for peripheral use. The catheter effectively reduced the thrombus burden in all cases, achieving satisfactory final angiographic results. One case of no-reflow was observed following lesion dilatation prior to thrombectomy, but no other major in-hospital adverse events occurred. At mid-term follow-up, all patients remained free from angina. These preliminary findings suggest that this approach could represent a promising option for managing highly thrombotic coronary lesions, but further studies with larger populations and long-term follow-up are needed to confirm these results.

## 1. Introduction

Percutaneous coronary intervention (PCI) of highly thrombotic coronary lesions is challenging. They are more frequent in an acute coronary syndromes (ACS) setting, especially ST-segment elevation myocardial infarction (STEMI), and are linked to a higher risk of procedural complications and worse long-term clinical outcomes [1].

In this setting, coronary thrombus aspiration can be an option. Even if routine use of thrombectomy in ACS PCI is not indicated, in selected cases with high-thrombus burden it may be considered [2].

Several devices are currently available for coronary thrombus aspiration and can be divided into manual-based and mechanical-based aspiration systems.

The Indigo™ System CAT™ RX catheter (Penumbra Inc., Alameda, CA, USA) is a mechanical-based device. It has been recently approved for coronary thrombectomy and clinical experience with this system is still limited [3].

The AngioJet Spiroflex^®^ (Boston Scientific, Marlborough, MA, USA), a coronary mechanical rheolytic thrombectomy (RT) catheter, is no longer available in Europe for commercial reasons. However, the AngioJet Solent^®^ Dista (Boston Scientific, Marlborough, MA, USA), the AngioJet catheter for peripheral arteries thrombus aspiration, is currently accessible.

In this report, we present six high-thrombus-burden STEMI patients treated by PCI and the AngioJet Solent^®^ Dista catheter.

## 2. Case Series

This case series consists of patients with STEMI and high-thrombus burden who were treated with RT at our center. Patients’ characteristics and periprocedural outcomes are summarized in Table 1.

### 2.1. Case 1

**Figure 1:**
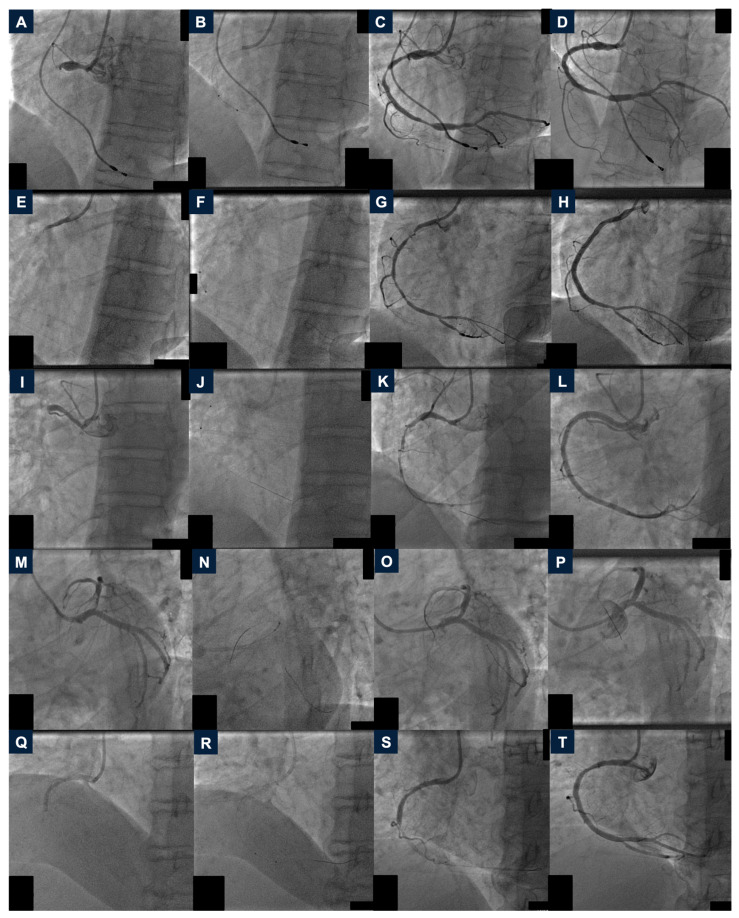
Percutaneous coronary interventions of the clinical cases from 1 to 5. Case 1, I line. (**A**): Baseline angiography demonstrating a high thrombus burden occlusion of the proximal right coronary artery (RCA). (**B**): Rheolytic thrombectomy (RT) with the AngioJet Solent^®^ Dista catheter. (**C**): Angiography after RT. (**D**): Final coronary angiography. Case 2, II line. (**E**): Baseline angiography showing a highly thrombotic occlusion of proximal RCA. (**F**): RT with the Solent^®^ Dista catheter. (**G**): Angiography after thrombectomy. (**H**): Final result. Case 3, III line. (**I**): Baseline coronary angiography. (**J**): Thrombus aspiration with the Solent^®^ Dista catheter. (**K**): Angiography after mechanical thrombectomy. (**L**): Final angiography. Case 4, IV line. (**M**): Baseline angiography showing a high-thrombus burden sub-occlusive stenosis of the ostial left anterior descending artery. (**N**): RT with the AngioJet Solent^®^ Dista catheter. (**O**): Angiography after RT. (**P**): Final result. Case 5, V line. (**Q**): Baseline angiography demonstrating a highly thrombotic occlusion of the mid RCA. (**R**): RT. (**S**): Angiography after RT. (**T**): Final angiographic result. RCA: right coronary artery; RT: rheolytic thrombectomy.

In January 2024, a 65-year-old Caucasian woman with a pacemaker and history of breast cancer was admitted to our unit for STEMI. Emergent coronary angiography revealed a dominant right coronary artery (RCA), with a highly thrombotic occlusion in the proximal part (Figure 1, panel A). Weight- and activated clotting time (ACT)-based unfractionated heparin (UFH) and Ticagrelor 180 mg were administered (in our hub center and the connected spoke centers, the first UFH dose and Acetylsalicylic acid loading dose are always given upon STEMI diagnosis). PCI was started with RT and the AngioJet Solent^®^ Dista catheter. Thrombolysis in myocardial infarction (TIMI) flow grade 3 was restored (Figure 1, panel B and C). The procedure was then completed with one drug-eluting stent (DES) implantation and adequate post-dilatation (Figure 1, panel D). No glycoprotein IIb/IIIa (GPIIb/IIIa) inhibitors were administered. The patient had no procedural or in-hospital complications, she was discharged on the fifth post-primary PCI day with 50% left ventricular ejection fraction (LVEF). The patient is free from angina at the one-year follow-up.

### 2.2. Case 2

In January 2024, a 57-year-old man with hyperlipidemia was admitted for sub-acute STEMI. Coronary angiography showed a highly thrombotic occlusion of the proximal RCA (Figure 1, panel E). Antithrombotic therapy with weight- and ACT-based UFH and Cangrelor was initiated. The AngioJet Solent^®^ Dista catheter restored the coronary perfusion, and two overlapping DES were delivered with good final results (Figure 1, panel F–H). No GPIIb/IIIa agents were administered. The patient had no in-hospital adverse events and was discharged on the fifth postoperative day with an LVEF of 45%. The patient had no clinical events and is free from angina at the one-year follow-up.

### 2.3. Case 3

In February 2024, a 63-year-old man was admitted to our unit for sub-acute STEMI. Coronary angiography revealed a highly thrombotic occlusion of the proximal RCA (Figure 1, panel I). After administering weight- and ACT-based UFH and Prasugrel 60 mg, RT with the Solent^®^ Distal catheter was performed, and four overlapping DES were implanted. The final result was good with TIMI flow grade 3 (Figure 1, panel J–L and Video S1). No GPIIb/IIIa agents were infused. The patient had no procedural and in-hospital adverse events. He was discharged on the fifth post-primary PCI day with an LVEF of 45%. The patient is free from angina at the one-year follow-up.

### 2.4. Case 4

In April 2024, a 61-year-old woman was admitted for STEMI. Emergent coronary angiography showed a highly thrombotic sub-occlusive stenosis of ostial left anterior descending (LAD) with TIMI flow grade 1 (Figure 1, panel M). Antithrombotic therapy with weight- and ACT-based UFH and Cangrelor was initiated. Mechanical thrombectomy with the AngioJet Solent^®^ Dista catheter was performed, restoring TIMI flow grade 3 (Figure 1, panel N and O). PCI was completed by delivering one DES on the left main coronary artery-LAD and adequate post-dilatation (Figure 1, panel P and Video S2). No GPIIb/IIIa inhibitors were administered. The patient had no procedural or in-hospital complications and she was discharged on the fifth post-primary PCI day with an LVEF of 50%. She is free from angina at the ten-month follow-up.

### 2.5. Case 5

In May 2024, a 72-year-old woman with previous right lung cancer was admitted for sub-acute STEMI. Coronary angiography revealed mid-RCA highly thrombotic occlusion (Figure 1, panel Q). Weight- and ACT-based UFH and Ticagrelor 180 mg were administered. RT with the Solent^®^ Dista catheter was performed, followed by the implantation and post-dilatation of one DES. The final TIMI flow grade was 3 and no complications were reported (Figure 1, panel R–T). No GPIIb/IIIa agents were infused. The patient was discharged on the third postoperative day with an LVEF of 52%. The patient had no adverse events and is free from angina at the eight-month follow-up.

### 2.6. Case 6

**Figure 2:**
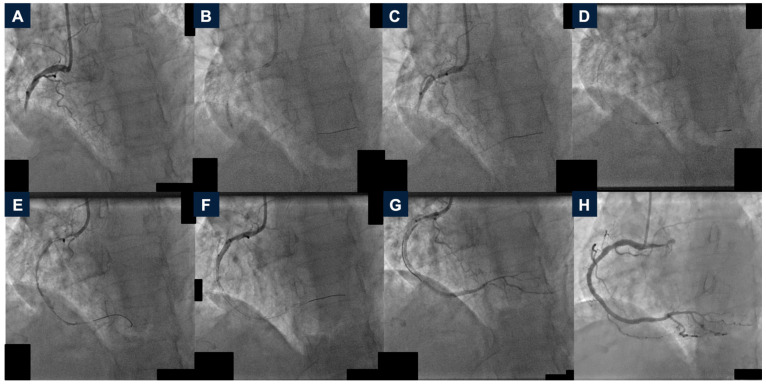
Case 6 procedure and angiographic follow-up. (**A**): Baseline angiography showing a high-thrombus-burden occlusion of the mid right coronary artery. (**B**): Lesion dilatation with a semi-compliant balloon. (**C**): Angiography after lesion dilatation demonstrating no coronary flow. (**D**): Mechanical thrombus aspiration with AngioJet Solent^®^ Dista catheter. (**E**): Coronary angiography after mechanical thrombectomy showing a thrombolysis in myocardial infarction (TIMI) flow grade 1. (**F**): Angiography after drug-eluting stent implantation, complicated by no-reflow phenomenon. (**G**): Angiographic result after intra-coronary Adenosine and Nitrates administration, TIMI final flow grade 1. (**H**): Two-month angiographic follow-up, TIMI flow grade 3. TIMI: thrombolysis in myocardial infarction.

In May 2024, a 77-year-old woman with chronic kidney disease and dialysis treatment, severe mitral regurgitation, and Parkinson disease was admitted to our unit for sub-acute STEMI. She had a highly thrombotic occlusion of the mid-RCA (Figure 2, panel A). After administration of weight- and ACT-based UFH and Cangrelor, PCI was initiated with balloon dilatation, but this was ineffective in restoring peripheral coronary perfusion (Figure 2, panel B and C). Therefore, RT using the AngioJet Solent^®^ Dista catheter was performed, successfully recanalizing the vessel with a TIMI flow grade of 1 (Figure 2, panel D and E). Lesion pre-dilatation was completed, and two overlapping DES were implanted. The procedure was complicated by no-reflow, which was treated with intra-coronary administration of Adenosine and Nitrates. The final angiographic result was satisfactory with a TIMI final flow grade 1 (Figure 2, panel F and G). No GPIIb/IIIa inhibitors were administered. No in-hospital adverse events were reported, and the patient was discharged on the seventh postoperative day with a LVEF of 45%. A staged-PCI was planned after two months due to a critical proximal LAD stenosis. The two-month angiographic follow-up showed a good-RCA PCI result and the TIMI flow grade was 3 (Figure 2, panel H and Video S3). After LAD PCI, the patient is free from angina at the six-month follow-up.

## 3. Discussion

PCI of highly thrombotic coronary stenoses is still a major concern for interventional cardiologists. Percutaneous treatment of these lesions is still associated with a higher complication rate, as a high thrombus burden is an independent risk factor for worse clinical outcomes [4].

In this complex setting, the role of thrombectomy has been investigated. Călburean et al. analyzed the long-term survival of STEMI patients treated with Ticagrelor, Eptifibatide, or manual thrombus aspiration. In their retrospective registry, manual thrombectomy failed to demonstrate a long-term survival benefit. Similarly, randomized clinical trials showed no significant clinical advantage of manual thrombectomy over PCI alone. Moreover, patients treated with manual thrombus aspiration had a higher risk of stroke at 30 days. That is why routine use of thrombectomy is not recommended in primary PCI [2,5,6,7,8].

Randomized trials reported in European guidelines have only investigated manual thrombus aspiration in STEMI. Manual thrombectomy catheters are syringe-based systems, and their vacuum force decreases during blood aspiration. This limitation can reduce the efficacy of thrombectomy during the procedure and increase the risk of thrombus embolization during catheter removal. These devices were investigated in STEMI patients, although thrombectomy could provide a procedural advantage over PCI alone in high-thrombus-burden ACS lesions [9].

In mechanical-based technology we have the Penumbra series and the AngioJet series catheters.

The Indigo™ System CAT™ RX catheter is the Penumbra device for coronary thrombectomy. It is a sustained mechanical thrombus aspirator, composed of the CAT™ RX catheter which is connected by an aspirator tubing to a Penumbra aspiration pump (Penumbra ENGINE series). The catheter is a rapid exchange system. The pump gives a continuous vacuum pressure up to 737 mmHg [3].

Clinical experience with the Penumbra device is limited.

Peng S. et al. assessed the CAT™ RX device performance in an all-comers population (83 patients, 85 lesions treated). In their retrospective registry the most common clinical presentation was ACS (92.7%). A final TIMI flow grade 3 was obtained in the 76% of cases. Technical success was achieved in the 88.9% of the cases treated by the CAT™ RX catheter. The rate of post-procedural stroke was 1.3%. At 30 days, the composite of cardiovascular death, recurrent myocardial infarction, cardiogenic shock, new or worsening New York Heart Association Class IV heart failure, and stroke, was 8.5% [10].

Sedhom R. et al. in their series investigated complication and CAT™ RX catheter failure in the Manufacturer and User Facility Device Experience (MAUDE) database. Their series included 127 reports. The most common failure modes were catheter damage and resistance during use (64.6% and 49.6%, respectively). The procedure was completed in 96.1% of cases. Stroke occurred in 1.6%, coronary dissection in 0.8%, and death in 2.4% [11].

The Indigo™ System CAT™ RX system in ACS has been studied in two small series. The final TIMI flow grade 3 ranged between 88% and 97.2%. No case of ischemic stroke was reported. Maximum cardiovascular death rate at 30 days was 1.4% [12,13].

The Penumbra coronary atherectomy catheter in ACS with high-thrombus burden has been evaluated in the ROPUST study and the CHEETAH study. In both registries, the final TIMI flow grade 3 rate exceeded 90% (90.2% and 97.5%, respectively). Major adverse cardiovascular events (MACE) rates were acceptable (8.9% at 6 months in the ROPUST study, 3.60% at 30 days in the CHEETAH study). No device-related serious adverse events were reported. Interestingly, in the ROPUST study, failure to achieve a TIMI grade 3 final flow was associated with balloon dilatation prior to thrombectomy. In our series, we had only one no-reflow case, when lesion dilatation preceded mechanical thrombus aspiration (case 6) [9,14].

The AngioJet technology is based on the Bernoulli effect. A high-speed saline jet moves retrogradely and creates a negative pressure zone with a strong and sustained vacuum effect (about −600 mmHg, 60 times greater than a manual thrombectomy catheter). The catheter distal part has some inflow and outflow doors. These holes allow thrombus fragmentation and aspiration. The saline pushes the clots removed into an external bag. The AngioJet Spiroflex^®^ and Solent^®^ Dista characteristics are summarized in Table 2. The Spiroflex^®^ catheter has a rapid exchange design. Instead, the Solent^®^ Dista is an over-the-wire system and needs a 300 cm guidewire (or a standard-length coronary guidewire with a guidewire extension). This can be quite cumbersome, especially in the STEMI setting. The Solent^®^ Dista has a lower diameter with a better crossing profile. It can be used in smaller vessels than Spiroflex^®^ (1.5 mm and 2 mm, respectively). This is a very important advantage, as in ACS with high-thrombus burden the peripheral part of the vessel is often not visible. The peripheral catheter also allows for the swapping of the guidewire, lytic, and contrast injection. The Spiroflex^®^ catheter has a higher flow rate than the Solent^®^ Dista (40 mL/min and 23 mL/min, respectively), but in our experience it does not affect clinical efficacy [15,16].

Clinical performance of rheolytic thrombectomy has been investigated in randomized trials and meta-analysis.

The JETSTENT trial is a multicenter, randomized, two arms study. It compared RT before direct stenting with direct stenting alone in STEMI patients and high-thrombus burden. Coprimary efficacy outcomes were ST-segment resolution at 30 min after PCI and 99mTc-sestaimibi scintigraphy infarct size at day 30. Clinical secondary endpoints were MACE, a composite of death, myocardial infarction, target vessel revascularization, and stroke at day 30, 6 months, and 1 year. Between December 2005 and September 2009, 501 patients were enrolled. In the AngioJet arm, subjects had a more frequent ST-segment resolution (RT 85.8% vs. direct stenting 78.8%—*p* = 0.043). The infarct size area did not vary between the two groups (11.8% with interquartile range (IQR) 3.15–23.70% vs. 12.7% with IQR 4.75–23.30%—*p* = 0.398). Migliorini A. et al. reported better secondary clinical outcome rates with RT at 6 months (11.2% vs. 19.4%—*p* = 0.011) and 1 year (14.9% vs. 22.7%—*p* = 0.036), while at day 30 the statistical significance was not reached (3.1% vs. 6.9%—*p* = 0.05) [17].

Navarese E. P. et al. compared mechanical thrombectomy with manual thrombus aspiration in STEMI. In their meta-analysis, they reviewed published studies between November 1994 and June 2013, examining both direct and indirect comparisons of manual thrombectomy, mechanical thrombus aspiration, and a placebo. Clinical outcomes investigated were mortality, re-infarction, and stroke at 30 days. Procedural endpoints were a final TIMI grade 3 flow and complete ST-segment resolution. Statistical assessed odds ratio (OR) with 95% confidence interval (CI). The direct analysis included two randomized trials and one non-randomized study (N = 513), while the indirect analysis comprised twenty-one non-randomized studies (N = 4514). The direct meta-analysis did not show any difference between the two technologies: the two groups had similar rates of mortality (OR (95% CI): 0.95 (0.51–1.78)—*p* = 0.88), re-infarction (0.91 (0.36–2.31)—*p* = 0.84), ST-segment resolution (1.17 (0.69–1.99)—*p* = 0.55), and final TIMI grade 3 flow (0.92 (0.62–1.37)—*p* = 0.69); stroke frequency was not evaluated due to the low number of data available. In the indirect comparison analysis, 30-day mortality was lower in the manual thrombectomy arm (0.51 (0.23–1.10)—*p* = 0.01), while re-infarction (1.47 (0.42–5.0)—*p* = 0.09) and stroke (1.57 (0.23–10.35)—*p* = 0.07) rates did not differ. When the trials with low-thrombus burden were excluded from the analysis, the two strategies had similar survival rates (0.88 (0.52–1.49)—*p* = 0.96), while re-infarction (2.41 (0.60–9.63)—*p* < 0.001) and stroke (1.58 (0.21–11.80)—*p* = 0.04) were significantly reduced by the mechanical thrombectomy technology. Procedural outcomes did not differ between the two strategies in the indirect analysis, even when the studies with low thrombus burden were excluded [18].

In a Bayesian meta-analysis, Grines C. L. et al. compared AngioJet to PCI alone in acute myocardial infarction patients. They analyzed myocardial infarction studies published from 1 January 1999, to 1 March 2007. OR with 95% CI for short-term mortality, short MACE, and post-procedural TIMI flow grade 3 were investigated. The RT group included 11 studies and 1018 subjects, while the PCI-alone data had 81 studies with 24076 patients. The two groups did not differ in mortality (OR 0.98, 95% CI 0.53–1.50), MACE (OR 1.25, 95% CI 0.54–2.40), and TIMI 3 final flow (OR 1.12, 95% CI 0.70–2.27). Remarkably, the AngioJet group had higher risk-profile patients, as it included more patients with large-thrombus burden, rescue PCI, and longer symptom duration before PCI [19].

In our center, we do not routinely perform manual thrombus aspiration in STEMI patients, as recommended in the European Guidelines. In 2024, we performed 202 STEMI PCI procedures, with manual thrombectomy used in only four cases (2%). These cases demonstrated sub-optimal thrombus removal, and in one instance the device failed to cross the lesion due to vessel bending.

In the same year, RT was instead used in six highly thrombotic STEMI lesions (all cases reported in this series) with satisfactory angiographic and clinical outcomes. In our series, the AngioJet Solent^®^ Dista catheter proved to be safe and effective in treating the six reported cases. It reduced the thrombus burden in all cases. Thanks to its low profile, the Solent^®^ Dista catheter was able to cross all the lesions, despite coronary bends and calcifications. We had only one no-reflow case (case 6). In this instance, RT was performed as a bailout after lesion dilatation and, as previously reported, lesion dilatation prior to mechanical thrombus aspiration is associated with an increased risk of no-reflow/slow-flow. Overall, the patients experienced no clinical adverse events and remained free from angina at follow-up.

Based on our experience, we suggest that RT could be a valuable option in high-thrombus burden lesions, especially in sub-acute STEMI. Even though the Solent^®^ Dista catheter is currently not approved for coronary use, in our series it effectively performed thrombectomy in six patients with no major in-hospital averse events. However, its use in highly thrombotic ACS coronary lesions needs to be systematically tested in larger studies with long-term follow-up.

Our report presents several limitations. First, it is not a clinical study, but a description of clinical cases performed in an emergency setting. In these cases, a device with CE certification for thrombectomy in human peripheral arteries was used for thrombus aspiration in coronary arteries. Only a small number of patients were treated in our series, with a mid-term clinical follow-up. Additionally, the majority of our patients were female and had a low-risk clinical profile (no previous cardiologic medical history, no diabetes, and no family history of coronary artery disease), which is somewhat uncommon in STEMI patients.

## 4. Conclusions

Percutaneous treatment of high-thrombus burden lesions is one of the most challenging scenarios for the interventional cardiologist due to the worse acute and long-term clinical outcomes.

The present report describes a case series of six highly thrombotic STEMI lesions treated with the AngioJet Solent^®^ Dista catheter, an RT catheter designed for peripheral use. In these cases, the device demonstrated the ability to reduce the thrombus burden and, after completing the PCI, achieve satisfactory final angiographic results and favorable clinical outcomes.

Further studies with larger patient populations and extended long-term follow-ups are required to better evaluate these findings and assess the broader applicability of this approach.

## Figures and Tables

**Table 1:** Treated patients’ characteristics and periprocedural outcomes.

Demographic	
Age (mean)	66
Sex (male, %)	33.3
Comorbidities	
Diabetes mellitus (%)	16.7
Hypertension (%)	16.7
Hyperlipidemia (%)	66.7
Family history CAD (%)	0
Current smoker (%)	33.3
Chronic kidney disease (%)	16.7
Prior MI (%)	0
Prior PCI (%)	0
Prior CABG (%)	0
Prior stroke (%)	0
Clinical indications	
NSTEMI (%)	0
STEMI (%)	100
Antithrombotic therapy prior primary PCI	
UFH (%)	100
Acetylsalicylic acid (%)	100
Ticagrelor (%)	0
Prasugrel (%)	0
Clopidogrel (%)	0
Antithrombotic therapy during primary PCI	
UFH (%)	100
Cangrelor (%)	50
Ticagrelor (%)	33.3
Prasugrel (%)	16.7
Clopidogrel (%)	0
GPIIb/IIIa inhibitors (%)	0
Procedural data	
Target lesion RCA (%)	83.3
Target lesion LAD (%)	16.7
Target lesion LCx (%)	0
Target lesion RI (%)	0
Target lesion LMCA (%)	0
Pre-PCI TIMI flow	
0 (%)	83.3
1 (%)	16.7
2 (%)	0
3 (%)	0
Moderate/severe calcification (%)	50
Moderate/severe proximal tortuosity (%)	83.3
RT as first intention strategy (%)	83.3
RT performed in bail-out (%)	16.7
Pre-dilatation (%)	16.7
N. DES implanted (mean)	2
Post-dilatation (%)	50
Post-PCI TIMI flow	
0 (%)	0
1 (%)	16.7
2 (%)	0
3 (%)	83.3
Periprocedural outcome	
No-reflow/slow-flow phenomenon (%)	16.7
Stent thrombosis (%)	0
Cardiogenic shock (%)	0
Cardiovascular death (%)	0
Stroke (%)	0

CABG: coronary artery bypass graft; CAD: coronary artery disease; DES: drug eluting stent; GPIIb/IIIa: glycoprotein IIb/IIIa; LAD: left anterior descending; LCx: left circumflex; LMCA: left main coronary artery; MI: myocardial infarction; NSTEMI: non-ST-segment elevation myocardial infarction; PCI: percutaneous coronary intervention; RCA: right coronary artery; RI; ramus intermedius; RT: rheolytic thrombectomy; STEMI: ST-segment elevation myocardial infarction; TIMI: thrombolysis in myocardial infarction; UFH: unfractioned heparin.

**Table 2:** AngioJet Spiroflex^®^ and Solent^®^ Dista catheters characteristics.

AngioJet Catheter	Indications Approved	Delivery Platform	Minimum Vessel Diameter	Catheter Length	Catheter Diameter	Guidewire	Introducer Sheath	Contrast/Lytic Injection Port	Guidewire Swappable	Flow Rate	Maximum Run Times (Total Run Time/Run Time with Blood Flow)
Spiroflex^®^	Coronary arteries, saphenous vein grafts, peripheral arteries	rapid exchange	2 mm	135 cm	4 F	0.014 in	5 F (6 F with a guide catheter)	No	No	40 mL/min	600 s/300 s
Solent^®^ Dista	Peripheral arteries	over-the-wire	1.5 mm	145 cm	4 F/3 F	0.014 in	4 F	Yes	Yes	23 mL/min	600 s/300 s

## Data Availability

The original contributions presented in this study are included in the article/Supplementary Materials. Further inquiries can be directed to the corresponding author.

## Supplementary Materials

The following supporting information can be downloaded at: https://www.mdpi.com/article/10.3390/jcdd12020072/s1, Video S1: Case 3 procedure: Diagnostic angiography demonstrated a high thrombus burden sub-acute occlusion of the proximal right coronary artery. After rheolytic thrombectomy, four overlapping drug-eluting stents were implanted with good final results. Video S2: Case 4 primary percutaneous coronary intervention: Diagnostic angiography demonstrated a highly thrombotic lesion of the ostial left anterior descending artery. The stenosis was treated with an AngioJet Solent^®^ Dista catheter and the delivery of a drug-eluting stent on the left main coronary artery-left anterior descending. Final angiographic result was good. Video S3: Case 6 procedure and two-month follow-up: Diagnostic angiography showed a high thrombus burden occlusion of the mid right coronary artery. Lesion dilatation was performed but was ineffective in restoring coronary flow. Rheolytic thrombectomy with an AngioJet Solent^®^ Dista catheter was able to recanalize the vessel. After completing lesion pre-dilatation, two overlapping drug-eluting stents were implanted. The procedure was complicated by no-reflow, which was treated with intra-coronary Adenosine and Nitrates administration. The final angiographic result was satisfactory. The two-month angiographic follow-up showed good result of the right coronary artery intervention with a thrombolysis in myocardial infarction flow grade of 3.
